# Trends in functional disability and cognitive impairment among the older adult in China up to 2060: estimates from a dynamic multi-state population model

**DOI:** 10.1186/s12877-021-02309-4

**Published:** 2021-06-22

**Authors:** John P. Ansah, Chi-Tsun Chiu, Aloysius Chia Wei-Yan, Tessa Lui Shi Min, David B. Matchar

**Affiliations:** 1 Duke-NUS Medical School, Singapore, Singapore; 2 Institute of European and American Studies Academia Sinica, Taipei, Taiwan

## Abstract

**Background:**

Available evidence suggests that cognitive impairment (CI), which leads to deficits in episodic memory, executive functions, visual attention, and language, is associated with difficulties in the capacity to perform activities of daily living. Hence any forecast of the future prevalence of functional disability should account for the likely impact of cognitive impairment on the onset of functional disability. Thus, this research aims to address this gap in literature by projecting the number of older adults in China with functional disability and cognitive impairment while accounting for the impact of cognitive impairment on the onset of functional disability.

**Methods:**

We developed and validated a dynamic multi-state population model which simulates the population of China and tracks the transition of Chinese older adults (65 years and older) from 2010 to 2060, to and from six health states—(i) active older adults without cognitive impairment, (ii) active older adults with cognitive impairment, (iii) older adults with 1 to 2 ADL limitations, (iv) older adults with cognitive impairment and 1 to 2 ADL limitations, (v) older adults with 3 or more ADL limitations, and (vi) older adults with cognitive impairment and 3 or more ADL limitations.

**Results:**

From 2015 to 2060, the number of older adults 65 years and older in China is projected to increase, of which the number with impairment (herein referred to as individuals with cognitive impairment and/or activity of daily living limitations) is projected to increase more than fourfold from 17·9 million (17·8–18·0) million in 2015 to 96·2 (95·3–97·1) million by 2060. Among the older adults with impairment, those with ADL limitations only is projected to increase from 3·7 million (3·6–3·7 million) in 2015 to 23·9 million (23·4–24·6 million) by 2060, with an estimated annual increase of 12·2% (12·1–12·3); while that for cognitive impairment only is estimated to increase from 11·4 million (11·3–11·5 million) in 2015 to 47·8 million (47·5–48·2 million) by 2060—this representing an annual growth of 7·07% (7·05–7·09).

**Conclusion:**

Our findings suggest there will be an increase in demand for intermediate and long-term care services among the older adults with functional disability and cognitive impairment.

**Supplementary Information:**

The online version contains supplementary material available at 10.1186/s12877-021-02309-4.

## Background

During the past few decades, the world has seen a rapidly ageing population in both developing and developed countries, due to declining fertility and mortality rates. China, with the largest population of older adults in the world, is rapidly ageing [1]. From 2007 to 2017, the number of persons in China aged 65 years and older increased from 106·36 million (representing 8·1% of the total population) to 158·31 million (representing 11·4% of the total population) [2]. According to the World Health Organization (WHO), it is estimated that by 2050, the population of China aged 60 and older will reach 35·1% [3]. The rapid growth in the number of older adults in China is a source of concern for policymakers due to the health and social care implications of aging. Aging could lead to undesirable outcomes such as rising dependency and caregiver burden; increased health care utilization for both acute and long-term care; and escalating healthcare cost [4–10].

While a fraction of the population is increasingly avoiding fatal events due to changes in lifestyle which modify the risk factors for mortality, thus delaying the age-at-onset and progression of diseases, the majority are not avoiding the physiological changes associated with aging and the accumulation of chronic conditions such as cognitive impairment and functional disability [11–16]. The rapid socio-economic development and strict implementation of family planning policies since 1980 has affected the structure of families in China, resulting in the erosion of the traditional family support for older persons [17]. This has led to increased demand for long-term care services from society and the government. Recently, the Chinese government has responded to the huge challenge of caring for the older adult by proposing policies aimed at increasing the supply and access to community-based older adult care services [18].

To plan and provide long-term care services needed in China, health and social services policy-makers responsible for planning the intermediate and long-term care services for older adults require an evidence-based and credible forecast of the current and future number of older Chinese adults with functional disability and cognitive impairment needing assistance to inform policy decisions. Projections of cognitive impairment and functional disability often involve simple extrapolation [19, 20] that fail to account for the transition across different health states; when transition rates are accounted for [21–24], they fail to consider the effect cognitive impairment have on the development of functional disabilities. Available evidence suggests that cognitive impairment, which leads to deficits in episodic memory, executive functions, visual attention, and language [25], is associated with difficulties in the capacity to perform activities of daily living [26]; hence any forecast of the future prevalence of functional disability should account for the likely impact of cognitive impairment/dementia on the onset of functional disability. Thus, this research aims to address this gap in literature by projecting the number of older adults in China with cognitive impairment and functional disability up to 2060, while simultaneously accounting for the impact of cognitive impairment on the onset of functional disability—measured herein by limitations in activities of daily living. The evidence-based projections from this research could help support planning for the number of older adults with care needs, infrastructural capacity required to meet the care needs of older adults, and human resources required to provide care services for older adults in China. Although this study focuses on China, the general insights presented herein are potentially useful to other countries undergoing a similar demographic transition, including Japan, South Korea, Taiwan, India, and Singapore.

## Methods

### Model design

In order to project the number of older adults in China with cognitive impairment and functional disability, we developed and validated a dynamic multi-state population model [27, 28] which simulates the population of China and tracks the transition of older Chinese adults 65 years and older from 2010 to 2060, to and from six health states—(1) active older adult without cognitive impairment (where active is older adults with no ADL limitation), (2) active older adult with cognitive impairment, (3) older adult with 1 to 2 ADL limitations, (4) older adult with cognitive impairment and 1 to 2 ADL limitations, (5) older adult with 3 or more ADL limitations, and (6) older adult with cognitive impairment and 3 or more ADL. Within each health state, the population was further divided into two-dimensional vector: age (from age 65–100 and older) and gender (male and female). To ensure that our model is consistent with the population of China, an additional state which accounts for the population below age 65 is included; each year, the population becoming 65 years was assumed to enter the active older adult without cognitive impairment health state. Not unlike the older adult population, the population below age 65 was subdivided by age (age 0–64) and gender. The population below age 65 increases via births and migration and decreases via deaths, emigration and becoming age 65. Births was estimated using female reproductive age and fertility rates; deaths were obtained from life tables. At the end of each year, the surviving population in each age cohort flows to the subsequent cohort, with the exception of the final age cohort—age 100 and older. The health states with cognitive impairment are further divided into three categories—mild, moderate and severe—cognitive impairment with age-specific, gender-specific and health state specific transition rates accounting for the movements across cognitive impairment categories; the cognitive impairment transition rates are reported in earlier publication in the reference as cited [28]. Transition across health states is determined by 1-year age-specific, gender-specific and health state specific transition rates.

### Health states

Cognitive function of the older adults was measured using the Chinese version of Mini Mental State Examination (MMSE), consisting of 30 items [29, 30]. MMSE scores range from 0 to 30; higher scores indicate better cognition. Participants’ orientation, memory, attention, calculation, language, and written and visual construction are assessed in the MMSE. Cognitive function was classified as follows: intact (MMSE score ≥ 24), mild cognitive impairment (18–23), moderate cognitive impairment (10–17), and severe cognitive impairment (≤9) [28, 31]. Functional status was measured using Activity of Daily Living (ADL) consisting of taking a bath/shower, dressing up, eating, standing up from or sitting on chair, walking around the house, and using the toilet. Those who reported no limitations in any of these activities were classified as active older adult; those with 1 or 2 ADL limitations were classified as older adult with 1 to 2 ADL limitations. Lastly, those with 3 or more ADL limitations were classified as older adult with 3 or more ADL limitations. Thus, active older adult without cognitive impairment is defined herein as older adults who have no ADL limitations and are cognitively intact. Active older adult with cognitive impairment is defined as older adults with no ADL limitations but have cognitive impairment—i.e., either mild, moderate, or severe cognitive impairment.

### Model assumptions

Fertility rate from 2010 to 2017 was used, and the 2017 fertility rate was assumed to remain constant throughout the projection because a change in fertility rate will have no impact on the older adult population by 2060. On mortality, a future decline of 1·5% per annum was assumed [32]. Lastly, a 1% annual improvement in transition rates—based on authors’ estimates—across all health states were assumed to account for future advancement in behavioural and pharmacological interventions.

### Data and estimation of transition probabilities

Data from the 2012 and 2014 waves of the Chinese Longitudinal Healthy Longevity Survey (CLHLS) was used to estimate the transition rates across health states. The CLHLS is a longitudinal survey established in 1998, with baseline and follow-up surveys conducted in half of the counties and cities in the selected 22 provinces in China. This survey collects data on demographics, socioeconomic, lifestyle and dietary behaviours, health status, diseases, cognitive function, and physical performance. Further information regarding the CLHLS can be found in the source as cited [33]. The 2012 and 2014 waves were selected because they were follow-up surveys of closed cohort (i.e., cohort with fixed membership. Once the cohort is defined by enrolling subjects and follow up begins, no one can be added to the cohort), as compared to other waves. The survey consists of 1824 Chinese older adults (814 men and 1010 women) age 65 years and older. Demographic data—fertility rate, mortality rate, and initial population distribution—used to initialize the model was obtained from the National Bureau of Statistics in China. Functional disability was measured using ADL limitations and cognitive function by the Chinese version of the MMSE.

Using the 2012 and 2014 waves of CLHLS, the rates of transitioning from one cognitive state to another or death (with the exception of improvement in cognitive states from both moderate and severe CI) as well as transitioning from one functional status to another (with the exception of improvement from 3 and more ADL limitations) or death was estimated for the overall sample [34, 35]. In order to estimate 1-year transition probabilities from two waves of survey conducted over a 2 year gap (2012 and 2014), we employed a method adapted from the SAS code of a study by Cai et al., similar to that used in a previous publication estimating older persons with cognitive impairment in China [28, 36]. By assigning a cognitive state/functional status to the participants in year 2013, we were able to circumvent the lack of data in the year 2013 with minimal assumptions. To ensure comparability with an earlier study by Ansah et al. [28], the ensuing rules were applied: (1) If a participant had the same cognitive state/functional status in both 2012 and 2014, we assume he/she has been in that particular cognitive state/functional status in 2013 since there is no information on transition from the survey; and (2) If a participant was in different cognitive states/functional status in 2012 and 2014, then the transition is assumed to happen randomly between 2012 and 2014 (i.e. cognitive state/functional status in 2013 can be the same as that in 2012: transition happened in the end of 2013 or the beginning of 2014; or cognitive state/functional status in 2013 can be the same as that in 2014: transition happened in the end of 2012 or the beginning of 2014) [28]. The Eq. 1 below, with age and sex as covariate, was used to estimate the transition rate for cognitive state [28]. The equation was solved with multinomial logistic regression models using the “multinom” function in R (v3.2.1) [28].

1 $$ \ln \left(\frac{p_{ij}}{p_{ii}}\right)={\beta}_{0 ij}+{\beta}_{1 ij}\bullet age+{\beta}_{2 ij}\bullet {age}^2+{\beta}_{3 ij}\bullet Sex\dots \dots \dots $$

where p_ij_ is the transition rate from the current state *i* to state *j* (*i* ≠ *j*), where *i* corresponds to intact, mild, moderate, or severe cognitive impairment and *j* corresponds to the same states as well as death [28]. Transition rates were disaggregated according to age (single age cohort from age 65–100 and older) and gender (female, male) [28].

The Eq. 2 (with age, sex, and cognitive impairment status as covariate) was used to estimate the transition rate for functional disability. The equation was solved with multinomial logistic regression models using “multinom” function in R (v3.2.1).

2 $$ \ln \left(\frac{p_{ij}}{p_{ii}}\right)={\beta}_{0 ij}+{\beta}_{1 ij}\bullet age+{\beta}_{2 ij}\bullet sex+{\beta}_{3 ij}\bullet CI\dots \dots \dots $$

where p_ij_ is the transition rate from the current state *i* to state *j* (*i* ≠ *j*), where *i* corresponds to healthy, 1 to 2 ADL limitations, or 3 or more ADL limitations and *j* corresponds to the same states as well as death. Transition rates were disaggregated according to age (single age cohort from age 65–100 and older), gender (female, male), and cognitive impairment (no CI, with CI).

#### Model validation and sensitivity analysis

For the purpose of model validation, the simulation model was presented to demographers to verify its structure, assumptions, and model parameters used to initialize the model. In addition, we compared our model estimates on total population and older adult population with official estimates from the National Bureau of Statistics of China. Also, we compared our model estimates of the number of older adults with ADL limitations and cognitive impairment with that of available published estimates.

Following a similar process as Ansah et al. [28], the bootstrap method was used to estimate the likely distribution of transition rates to obtain the 95% confidence interval around the point estimates after computing point estimates for the transition rates from the multinomial logistic regressions. First, the sampling weights were rescaled to sum up to 100%. Using the “sample” function in R (v3.2.1), the weights were used as probabilities to draw respondents (by identification, ID) with replacement. In the sampling, each respondent (ID) may be drawn once, more than once or not at all. The process was repeated 1000 times to obtain 1000 datasets for the purpose of estimating the transition rates. Transition rates were estimated using the 1000 samples with the multinomial logistic regression model. The distribution of age and sex specific transition rates and 95% confidence intervals were obtained based on the 1000 sets of estimated transition rates. In order to obtain the likely variation in the projected number of older adults with cognitive impairment, transition rates from the bootstrap analysis were used as input to the sensitivity analysis.

## Results

### Transition rates by age, gender, and cognitive impairment

Fig S1 in the Additional file 1 shows the graphs of the transition rates by functional disability. For both sexes, the rate of transitioning to a worse functional disability state and death increased with age and cognitive impairment, while the rate of transitioning to an improved functional disability state decreased with age and without cognitive impairment. Transition rates from active to all health states, as well as that from one to two ADL to three or more ADL, and deaths including that of 3 or more ADL to deaths are higher for males than females. However, transition from one to two ADL to active was higher for females than males. In addition, similar with results from Ansah et al. [28] age and gender specific cognitive impairment rates of transition across cognitive states and death are presented in Fig. S2 in the Additional file 1. For both sexes, the transition rate to a worse cognitive state or death increased with age whereas the rate of transitioning to a better cognitive state decreased with age. In accordance with results from Ansah et al. [28], transition rates from intact to mild, moderate, or severe CI were higher in females than in males. Similar effects were found for transition rates from mild CI to intact or death, and from severe CI to death [28]. However, in contrast, transition rates from intact to death, mild CI to moderate or severe CI, and moderate CI to severe CI were higher for males than females. Lastly, the transition rate from moderate CI to death for males decreased to a level lower than females at very old age (about 97 years old) [28].

#### Projected number of older adult with functional disability and cognitive impairment

As shown in Table 1, based on our projections, the older adult population (individuals age 65 years and older) in China will increase on average 3·9% annually from 159·07 (158·98–159·16) million in 2015 to 439·34 (438·31–440·397) million by 2060. Of which the number with impairment —herein referred to as individuals with cognitive impairment and/or activity of daily living limitations—was projected to increase more than fourfold from 17·920 (17·829–18·012) million in 2015 to 96·184 (95·280–97·087) million by 2060. As a result, the proportion of the older adults with impairment was projected to increase from 11.2% (11.21–11.32) in 2015 to 21·83% (21·66–22·00) by 2060. On gender composition, the majority of the older adults with impairment were projected to be female (67·4% in 2015 and 52% by 2060); hence the proportion of the female older adults with impairment was projected to increase from 14·0% (13·9–14·1) in 2015 to 25·6% (25·4–25·8) by 2060 compared to 8·4%(8·3–8·5) in 2015, and 17·9% (17·7–18·1) in 2060 for the males. Though females form the majority of the impaired older adults, the rate of increase in impairment among the older adult males—estimated to be 501·2% (497·7–504·6) from 2015 to 2060; representing an annual increase of 11·1% — was projected to be higher than that of the females.

**Table 1:** Projected number of older adults with impairment (in millions) by gender by 95% uncertainty interval. Percentages are unadjusted

	2015	2060	Annual relative change (%)	Relative change (2015–2060)
**All older adult**				
Total older adult	159·07 (158·98–159·16)	439·34 (438·31–440·37)	3·92% (3·9–3·93)	176·1% (175·6–176·6)
Older adult without impairment	141·15 (141·04–141·26)	343·16 (342·44–343·88)	3·18% (3·17–3·19)	143·1% (142·7–143·4)
Older adult with impairment	17·920 (17·829–18·012)	96·184 (95·280–97·087)	9·71% (9·65–9·76)	436·7% (434·4–439)
Fraction with impairment (%)	11·26% (11·21–11·32)	21·83% (21·66–22·00)	2.08%(2.07–2.09)	93.6%(93.15–94.05)
**Female**				
Total older adult	81·175 (81·108–81·242)	221·45 (220·78–222·13)	3·84% (3·83–3·85)	172·8% (172·2–173·4)
Older adult without impairment	69·782 (69·709–69·856)	164·51 (164·09–164·93)	3·017% (3–3·025)	135·7% (135·4–136·1)
Older adult with impairment	11·393 (11·331–11.455)	56.943 (56.384–57.502)	8.88% (8.84–8.93)	399.8% (397.6–401.9)
Fraction with impairment (%)	14·03% (13·96–14·10)	25·62% (25·43–25·81)	1.84%(1.83–1.85)	82.61%(82.16–83.0)
**Men**				
Total older adult	77·90 (77·84–77·96)	217·88 (217·19–218·58)	3·99% (3·98–4·01)	179·6% (179–180·3)
Older adult without impairment	71·376 (71·300–71·452)	178·64 (178·11–179·18)	3·34% (3·33–3·35)	150·2% (149·8–150·7)
Older adult with impairment	6·527 (6·472–6·582)	39·241 (38·684–39·798)	11·14% (11·06–11·21)	501·2% (497·7–504·6)
Fraction with impairment (%)	8·38% (8·31–8·45)	17·92% (17·71–18·14)	2.53%(2.51–2.55)	113.84%(113.12–114.67)

As shown in Table 2, of the older adults with impairment, those 65–74 years was projected to increase from 3·180 (3·138–3·222) million in 2015 to 4·009 (3·949–4·069) million by 2060; that for the age cohort 75–84 was projected to be 1·5 times from 8·650 (8·599–8·700) million in 2015 to 22·329 (22·179–22·478) million by 2060. Not unlike the others, the number of older adults 85 years and older with impairment was projected to increase more than ten times from 6·090 (6·052–6·129) million in 2015 to 69·846 (68·993–70·699) million by 2060. According to our projections, by 2060, 72·6% of the older adults with impairment will be 85 years and older.

**Table 2:** Projected number of older adults with impairment (in millions) by gender and age cohorts

	2015	2060	Annual relative change (%)	Relative change (2015–2060)
**All older adult with impairment**				
65–74 years	3·180 (3·138–3·222)	4·009 (3·949–4·069)	0·579% (0·574–0·584)	26·06% (25·83–26·29)
75–84 years	8·650 (8·599–8·700)	22·329 (22·179–22·478)	3·514% (3·509–3·520)	158·15% (157·92–158·38)
85 and Over	6·090 (6·052–6·129)	69·846 (68·993–70·699)	23·26% (23·11–23·41)	1046% (1039–1053)
**Female**				
65–74 years	1·863 (1·838–1·887)	2·346 (2·309–2·383)	0·577% (0·569–0·584)	25·95% (25·60–26·28)
75–84 years	5·404 (5·369–5·439)	13·425 (13·320–13·529)	3·298% (3·291–3·305)	148·41% (148·10–148·72)
85 and Over	4·126 (4·099–4·154)	41·172 (40·644–41·700)	19·95% (19·81–20·09)	897·8% (891·6–903·9)
**Men**				
65–74 years	1·317 (1·296–1·339)	1·663 (1·634–1·692)	0·583% (0·579–0·587)	26·23% (26·06–26·40)
75–84 years	3·245 (3·218–3·273)	8·904 (8·814–8·994)	3·875% (3·865–3·884)	174·36% (173·93–174·77)
85 and Over	1·964 (1·946–1·983)	28·674 (28·163–29·184)	30·22% (29·94–30·49)	1359% (1347–1372)

As indicated in Table 3, among the older adults with impairment, those with ADL limitations only was projected to increase from 3·679 (3·621–3·737) million in 2015 to 23·984 (23·397–24·571) million by 2060, with an estimated annual increase of 12·2% (12·1–12·3); while that for cognitive impairment only was estimated to increase from 11·443 (11·373–11·514) million in 2015 to 47·848 (47·456–48·239) million by 2060—representing an annual growth of 7·0% (7·0–7·1). Lastly, older adult individuals with ADL limitations and cognitive impairment were estimated to grow 17·1% (16·9–17·3) annually from 2·798 (2·759–2·836) in 2015 to 24·352 (23·7921–24·913) million by 2060. By 2060, 10·691 (10·397–10·985) million, 30·141 (29·869–30·414) million and 16·110 (15·711–16·510) million of the female older adults will have ADL limitations only, cognitive impairment only and ADL limitations and cognitive impairment respectively. Among the older adult males, the projected number of individuals with ADL limitations only, cognitive impairment only and ADL limitations and cognitive impairment was 13·293 (12·896–13·689) million, 17·706 (17·470–17·943) million and 8·242 (7·960–8·523) million, respectively.

**Table 3:** Projected number of older adults with impairment (in millions) by type and gender

	2015	2060	Annual relative change (%)	Relative change (2015–2060)
**All older adult with impairment**				
ADL only	3·679 (3·621–3·737)	23·984 (23·397–24·571)	12·2% (12·1–12·3)	551·9% (546·1–557·5)
CI only	11·443 (11·373–11·514)	47·848 (47·456–48·239)	7·07% (7·05–7·09)	318·1% (317·2–318·9)
CI and ADL	2·798 (2·759–2·836)	24·352 (23·791–24·913)	17·1% (16·9–17·3)	770·4% (762·2–778·4)
**Female**				
ADL only	1·650 (1·623–1·676)	10·691 (10·397–10·985)	12·18% (12–12·34)	548·1% (540·5–555·4)
CI only	7·728 (7·674–7·782)	30·141 (29·869–30·414)	6·45% (6·43–6·46)	290% (289·2–290·8)
CI and ADL	2·016 (1·984–2·047)	16·110 (15·711–16·510)	15·5% (15·3–15·7)	699·3% (691·7–706·6)
**Men**				
ADL only	2·029 (1·989–2·070)	13·293 (12·896–13·689)	12·3% (12·1–12·4)	555% (548·5–561·2)
CI only	3·716 (3·678–3·754)	17·706 (17·470–17·943)	8·37% (8·33–8·4)	376·5% (375–378)
CI and ADL	0·782 (0·766–0·798)	8·242 (7·960–8·523)	21·2% (20·8–21·5)	953·8% (938·6–968·5)

The disaggregation of impairment by type and age cohorts, as indicated in Table 4, shows that majority of the older adult with impairment, irrespective of type, was observed among the older adult 85 years and older. By 2060, older adult 85 years and older with ADL limitations only was projected to be 18·692 (18·141–19·243) million; while that for cognitive impairment only is 29·408 (29·083–29·732) million and ADL limitations and cognitive impairment is 21·764 (21·193–22·299) million. Among those with ADL limitations only, the older adult 85 years and older constitute 77·9%, while that for cognitive impairment only was 61·4%. Lastly, 89·3% of the older adult with cognitive impairment and ADL limitation are 85 years and older. Tables S1-S7 in the Additional file 1 provides supplementary projected results.

**Table 4:** Projected number of older adults with impairment (in millions) by type, gender, and age cohorts

	2015	2060	Annual relative change (%)	Relative change (2015–2060)
**65–74 Years**				
ADL only	0·934 (0·92–0·95)	1·129 (1·102–1·156)	0·46% (0·44–0·48)	20·8% (19·7–21·6)
CI only	2·076 (2·044–2·108)	2·717 (2·665–2·768)	0·69% (0·68–0·70)	30·8% (30·4–31·3)
CI and ADL	0·170 (0·165–0·176)	0·163 (0·157–0·170)	−0·09% (−0·1- -0·08)	−4·19% (−4·93- -3·49)
**75–84 Years**				
ADL only	1·598 (1·573–1·623)	4·163 (4·089–4·237)	3·57% (3·56–3·58)	160·5% (159·9–161·07)
CI only	6·003 (5·960–6·045)	15·723 (14·598–15·848)	3·60% (3·59–3·60)	161·9% (161·7–162·1)
CI and ADL	1·049 (1·031–1·067)	2·443 (2·398–2·488)	2·95% (2·94–2·96)	132·8% (132·5–133·1)
**85 and over**				
ADL only	1·147 (1·129–1·166)	18·692 (18·141–19·243)	33·9% (33·4–34·4)	1529% (1507–1550)
CI only	3·365 (3·337–3·392)	29·408 (29·083–29·732)	17·2% (17·1–17·25)	773% (771–776)
CI and ADL	1·578 (1·555–1·601)	21·764 (21·193–22·299)	28·4% (28·07–28·73)	1278% (1262–1292)

## Discussion

Our estimates are consistent with available evidence from literature on the future number of older Chinese adults with cognitive impairment and functional disability, due to population aging [24, 28]. Findings from our research suggest that the number of older Chinese adults with cognitive impairment and functional disability is projected to increase more than fourfold from 2015 to 2060; consequently, the proportion of the older adults with impairment is projected to rise significantly by 2060. Among those with impairment, the majority will be women aged 85 years and older and cognitive impairment is projected to contribute more to impairment than ADL limitation.

The projected increase in older adults with cognitive impairment and functional disability in China is due mainly to an aging population. As income per capita increases in China, coupled with increased educational attainment as well as rising access to healthcare services, mortality is expected to decrease, thus shifting the age distribution of the population. Based on our simulation model, the proportion of the older adults in China 85 years and older is projected to increase from 7% in 2015 to 32% by 2060; while that of older adults 65 to 74 years old is projected to decrease from 62% in 2015 to 35% by 2060. Since the prevalence of functional disability and cognitive impairment increases with age, as the proportion of older adult in China 85 years and older increases (from 7% in 2015 to 32% in 2060), the number of older adults with functional disability and cognitive impairment is expected to increase as well. However, though increased access to healthcare services and changes in lifestyle could modify the risk factors and delay the age-at-onset of functional disability and cognitive impairment at younger age (age 65 to 84 years), at an older age it is much more difficult to avoid these conditions due to physiological changes, hence the increase in the number of older adults with functional and cognitive disability.

**Fig. 1:**
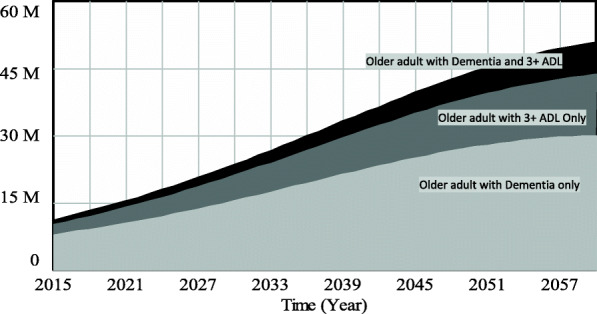
Projected number of older adult Chinese with nursing home type care needs

The finding that 21·8% of the older Chinese adults will develop impairment (functional disability or cognitive impairment) by 2060 of which the majority will be women aged 85 years and older, has policy implications for health and social care service needs. Evidence suggests that older adults with impairment are associated with greater informal care (as care hours provided by informal caregivers are much higher compared to those without impairment [37]), formal long-term care use [38, 39], and acute care utilization [40], and may result in growing health care expenditure [41]. Overall, this finding suggests that health and social care needs among the older adults in China are expected to increase significantly. Consequently, policy makers must be proactive in responding to these needs, lest unmet care needs among the older adult will increase leading to poor health outcomes. A delayed response to health and social care needs of the older adults could lead to longer waiting time for services, increased family/informal caregiving burden, and increased number of older adults in worse health states leading to increased health care cost. Further analysis indicates that among those with impairment, the number of older adults with nursing home type care needs (individuals with three or more ADL limitations or moderate to severe cognitive impairment or both) is forecasted to increase significantly—57·164 million (55·993–58·335 million) by 2060—as shown in Fig. 1. Among those with nursing home type care needs, the number with three or more ADL limitations and dementia—defined herein as individuals with moderate or severe cognitive impairment—is estimated to increase from 7·856 million (7·801–7·911 million) in 2015 to 31·109 million (30·745–31·472 million) by 2060. Also, the older adult with three or more ADL limitations only is projected to increase from 2·047 million (2·013–2·081 million) in 2015 to 15·999 million (15·527–16·472 million) by 2060, while that for older adult with dementia and three or more ADL limitations is estimated to increase from 1·101 million (1·081–1·122 million) in 2015 to 10·056 million (9·721–10·391 million) by 2060.

The emphasis of filial piety and family as the unit of care for the older adults in Asian culture suggests that older adults with functional disability and cognitive impairment are likely to be cared for at home (Liu and National University of Singapore, 1998). It is estimated that up to 96% of Chinese older adults with dementia are cared for by family members at home [42]. As the number of older adults with disability increases, care burden (especially informal care burden if current living arrangement remains unchanged) is expected to increase. The expected increase in informal care burden will put strain on family caregivers, exposing them to negative caregiving consequences such as depression, anxiety disorders, and weakened immunity [43]. Hence, policy makers should implement caregiver support systems for informal caregivers to facilitate caregiving and reduce the negative impact of caregiving.

The finding from this research emphasises the need for China to proactively develop its primary care sector to provide enhanced healthcare services for the rapidly aging population with chronic diseases such as cognitive impairment and functional disability. In addition, the capacity of social care services such as the recently proposed community-based older adult care services and nursing homes, needs to be ramped up and effectively linked to the healthcare system to provide integrated health and social care services in order to meet the needs of the older adults to enhance active aging. Policy makers should emphasize educating the general population on the likely increase of informal/family caregiving burden as the number of older adults with disability increase. Education programs will increase awareness of impairment associated with aging, and equip families with skills and knowledge on how to care for and manage individuals with cognitive impairment and functional disability.

Lastly, the findings of this study, especially the transition rates from different disability groups, could be used as baseline transition rates to evaluate the impact of World-Wide FINGER studies [44] on cognitive decline among the older adults in China. FINGER is the Finish multi-domain geriatric intervention study to prevent cognitive impairment and disability. Currently, there are similar ongoing studies around the world (such as US-POINTER, MIND-CHINA, SINGER in Singapore, and UK-FINGER) [42]. The value of the dynamic multi-state population model presented herein is its ability to use trial results from the MIND-CHINA multi-domain intervention and scale it to the population level to explore the health and economic benefits of the intervention.

The simulation model used for this study has several limitations. First, we assume that individuals turning 65 in China enter age 65 without any cognitive impairment or functional disability. Including the transition rate of cognitive impairment and functional disability among older adults becoming 65 is likely to increase marginally the projected number of individuals with impairment. Future models projecting trends in functional disability and cognitive impairment among older adults 65 years and older in China should consider including this transition to increase the accuracy of the projections. Second, we assume that future improvement in transition rates is fixed at 1% per year over the simulation time. This percentage may increase or decrease conditional on future advancement in behavioural and pharmacological interventions, thus changing the projections presented herein. Lastly, the estimate of transition rates across health states were derived from a fairly small CHLS datasets given the population of China. There is a need to establish the reliability of the estimated transition rates based on larger datasets to obtain robust projections.

## Conclusion

In conclusion, our evidence based multi-state population forecasting model, incorporating transition across different health states and the impact of increasing prevalence of cognitive impairment on functional disability, projects that the number of older Chinese adults with cognitive and functional impairment will increase significantly by 2060. This expected increase is due to population aging and the resulting shift in age distribution among the population. The expected rise in the burden of age-related impairment poses a significant challenge that requires urgent policy development to address both intended and unintended consequences. Our findings suggest that there will be an increase in demand for intermediate and long-term care services among the older adults with functional disability and cognitive impairment. Whether this demand is filled by the family, the private sector, or the government is an issue that policy makers should consider in planning for health and social care in China.

## Supplementary Information

**Additional file 1: S1 Fig.** Transition rates of functional disability health states. **S2 Fig**. Transition rates of cognitive impairment health states. **S1 Table.** Projected number of older adult in China (million). **S2 Table.** Projected number of active older adult (older adult with no functional disability or cognitive impairment) in China (million). **S3 Table.** Projected number of older adult with 1–2 ADL limitations (functional disability) in China (million). **S4 Table.** Projected number of older adult with 3 or more ADL limitations (functional disability) in China (million). **S5 Table.** Projected number of older adult with mild cognitive impairment in China (million). **S6 Table.** Projected number of older adult with moderate cognitive impairment in China (million). **S7 Table.** Projected number of older adult with severe cognitive impairment in China (million). **S8 Table.** Results of literature review on projection of functional and cognitive disability in China.

## Data Availability

The data for this study is available at Duke Center for the Study of Aging and Human Development. The link to access the datasets is https://sites.duke.edu/centerforaging/programs/chinese-longitudinal-healthy-longevity-survey-clhls/

## Abbreviations

ADL: Activity of Daily Living
WHO: World Health Organization
MMSE: Mini Mental State Examination
CLHLS: Chinese Longitudinal Healthy Longevity Survey
CI: Cognitive Impairment
ID: Identification
FINGER: Finish Geriatric Intervention Study to Prevent Cognitive Impairment and Disability
US-POINTER: U.S. Study to Protect Brain Health through Lifestyle Intervention to reduce Risk
MIND-CHINA: Multimodal Intervention to delay Dementia and Disability in Rural China
SINGER: Singapore Intervention Study to prevent Cognitive Impairment and Disability
UK-FINGER: UK Finish Geriatric Intervention Study to Prevent Cognitive Impairment and Disability

## References

[1] Yip W, Hsiao WC (2008). The Chinese health system at a crossroads. Health Aff (Millwood).

[2] China Statistical Yearbook (2018). National Bureau Statistics of China.

[3] United Nations, Department of Economic and Social Affairs, Population Division. Word Population Ageing 2017-Highlights. 2017. (ST/ESA/SER.A/397).

[4] Berlau DJ, Corrada MM, Kawas C (2009). The prevalence of disability in the oldest-old is high and continues to increase with age: findings from the 90+ study. Int J Geriatr Psychiatry.

[5] Jacobs JM, Maaravi Y, Cohen A, Bursztyn M, Ein-Mor E, Stessman J (2012). Changing profile of health and function from age 70 to 85 years. Gerontology..

[6] Nie JX, Wang L, Tracy CS, Moineddin R, Upshur RE (2008). Health care service utilization among the elderly: findings from the study to understand the chronic condition experience of the elderly and the disabled (SUCCEED project). J Eval Clin Pract.

[7] Vilpert S, Ruedin HJ, Trueb L, Monod-Zorzi S, Yersin B, Bula C (2013). Emergency department use by oldest-old patients from 2005 to 2010 in a Swiss university hospital. BMC Health Serv Res.

[8] de Meijer C, Koopmanschap M, TB DU, van Doorslaer E. (2011). Determinants of long-term care spending: age, time to death or disability?. J Health Econ.

[9] Gerdtham UG (1993). The impact of aging on health care expenditure in Sweden. Health Policy.

[10] Yang Z, Norton EC, Stearns SC (2003). Longevity and health care expenditures: the real reasons older people spend more. J Gerontol B Psychol Sci Soc Sci..

[11] Christensen K, Doblhammer G, Rau R, Vaupel JW (2009). Ageing populations: the challenges ahead. Lancet..

[12] Crimmins EM, Saito Y (1993). Getting better and getting worse: transitions in functional status among older Americans. J Aging Health.

[13] Lau RS, Johnson S, Kamalanabhan TJ (2012). Healthy life expectancy in the context of population health and ageing in India. Asia Pac J Public Health.

[14] Lynch SM, Brown JS, Taylor MG, Uhlenberg P (2009). Demography of disability. International handbook of population aging.

[15] Vaupel JW (2010). Biodemography of human ageing. Nature..

[16] Sakari R (2013). Mobility and its decline in old age: determinants and associated factors: University of Jyväskylä.

[17] Zeng Y, Hesketh T (2016). The effects of China's universal two-child policy. Lancet..

[18] Liu Z, Han L, Feng Q, Dupre ME, Gu D, Allore HG, Gill TM, Payne CF (2019). Are China’s oldest-old living longer with less disability? A longitudinal modeling analysis of birth cohorts born 10 years apart. BMC Med.

[19] Woo J, Ho SC, Yuen YK, Yu LM, Lau J (1996). An estimation of the functional disability burden in elderly Chinese age 70 years and over. Disabil Rehabil.

[20] Giles LC, Cameron ID, Crotty M (2003). Disability in older Australians: projections for 2006-2031. Med J Aust.

[21] Hanewald K, Li H, Shao AW (2019). Modelling multi-state health transitions in China: a generalised linear model with time trends. Ann Actuarial Sci.

[22] Rickayzen BD, Walsh DEP (2002). A multi-state model of disability for the United Kingdom: implications for future need for long-term care for the elderly. Br Actuar J.

[23] Yu HM, Yang SS, Gao JW, Zhou LY, Liang RF, Qu CY (2013). Multi-state Markov model in outcome of mild cognitive impairments among community elderly residents in mainland China. Int Psychogeriatr.

[24] Hu B (2019). Projecting future demand for informal care among older people in China: the road towards a sustainable long-term care system. Health Econ Policy Law.

[25] Weintraub S, Wicklund AH, Salmon DP (2012). The neuropsychological profile of Alzheimer disease. Cold Spring Harb Perspect Med.

[26] Oros RI, Popescu CA, Iova CA, Mihancea P, Iova SO (2016). The impact of cognitive impairment after stroke on activities of daily living. HVM Int J Bioflux Soc.

[27] Ansah JP, Malhotra R, Lew N, Chiu C-T, Chan A, Bayer S, Matchar DB (2015). Projection of young-old and old-old with functional disability: does accounting for the changing educational composition of the elderly population make a difference?. PLoS One.

[28] Ansah JP, Koh V, Chiu C-T, Chei C-L, Zeng Y, Yin Z-X, Shi XM, Matchar DB (2017). Projecting the number of elderly with cognitive impairment in China using a multi-state dynamic population model. Syst Dyn Rev.

[29] Folstein MF, Folstein SE, McHugh PR (1975). “Mini-mental state” a practical method for grading the cognitive state of patients for the clinician. J Psychiatr Res.

[30] Katzman R, Zhang M, Ouang Ya Q, Wang Z, Liu WT, Yu E (1988). A Chinese version of the mini-mental state examination; impact of illiteracy in a Shanghai dementia survey. J Clin Epidemiol.

[31] Tombaugh TN, McIntyre NJ (1992). The mini-mental state examination: a comprehensive review. J Am Geriatr Soc.

[32] Banister J, Hill K (2004). Mortality in China 1964-2000. Popul Stud (Camb).

[33] Gu D, Yi Z, Poston DL, Vlosky DA, Gu D (2008). General data quality assessment of the CLHLS. Healthy longevity in China: demographic, socioeconomic, and psychological dimensions.

[34] Kryscio RJ, Schmitt FA, Salazar JC, Mendiondo MS, Markesbery WR (2006). Risk factors for transitions from normal to mild cognitive impairment and dementia. Neurology..

[35] Tyas SL, Salazar JC, Snowdon DA, Desrosiers MF, Riley KP, Mendiondo MS, Kryscio RJ (2007). Transitions to mild cognitive impairments, dementia, and death: findings from the Nun study. Am J Epidemiol.

[36] Cai L, Hayward M, Saito Y, Lubitz J, Hagedorn A, Crimmins E (2010). Estimation of multi-state life table functions and their variability from complex survey data using the SPACE program. Demogr Res.

[37] Ansah JP, Matchar DB, Love SR, Malhotra R, Do YK, Chan A (2013). Simulating the impact of long-term care policy on family eldercare hours. Health Serv Res.

[38] Liu K, Manton KG, Aragon C (2000). Changes in home care use by disabled elderly persons: 1982-1994. J Gerontol B Psychol Sci Soc Sci.

[39] Spillman BC, Pezzin LE (2000). Potential and active family caregivers: changing networks and the “sandwich generation”. Milbank Q.

[40] Wu CY, Hu HY, Li CP, Fang YT, Huang N, Chou YJ (2013). The association between functional disability and acute care utilization among the elderly in Taiwan. Arch Gerontol Geriatr.

[41] Spillman BC (2004). Changes in elderly disability rates and the implications for health care utilization and cost. Milbank Q.

[42] Dai B, Mao Z, Mei J, Levkoff S, Wang H, Pacheco M, Wu B (2013). Caregivers in China: knowledge of mild cognitive impairment. PLoS One.

[43] Schulz R, Martire LM (2004). Family caregiving of persons with dementia: prevalence, health effects, and support strategies. Am J Geriatr Psychiatr.

[44] Kivipelto ME, Martín N, Eg JJ (2018). World Wide Fingers will advance dementia prevention. Lancet Neurol.

