# Supplementary material for: Trends in functional disability and cognitive impairment among the older adult in China up to 2060: estimates from a dynamic multi-state population model
Source: BMC Geriatr. 2021 Jun 22;21:380. doi: 10.1186/s12877-021-02309-4 (PMC8218480; doi:10.1186/s12877-021-02309-4)
Supplement: Supplementary file 1 — Additional file 1: S1 Fig. Transition rates of functional disability health states. S2 Fig. Transition rates of cognitive impairment health states. S1 Table. Projected number of older adult in China (million). S2 Table. Projected number of active older adult (older adult with no functional disability or cognitive impairment) in China (million). S3 Table. Projected number of older adult with 1–2 ADL limitations (functional disability) in China (million). S4 Table. Projected number of older adult with 3 or more ADL limitations (functional disability) in China (million). S5 Table. Projected number of older adult with mild cognitive impairment in China (million). S6 Table. Projected number of older adult with moderate cognitive impairment in China (million). S7 Table. Projected number of older adult with severe cognitive impairment in China (million). S8 Table. Results of literature review on projection of functional and cognitive disability in China. [file 12877_2021_2309_MOESM1_ESM.docx]

**SUPPLEMENTATRY APPENDIX**

**RESULTS**

S1 Fig: Transition rates of functional disability health states


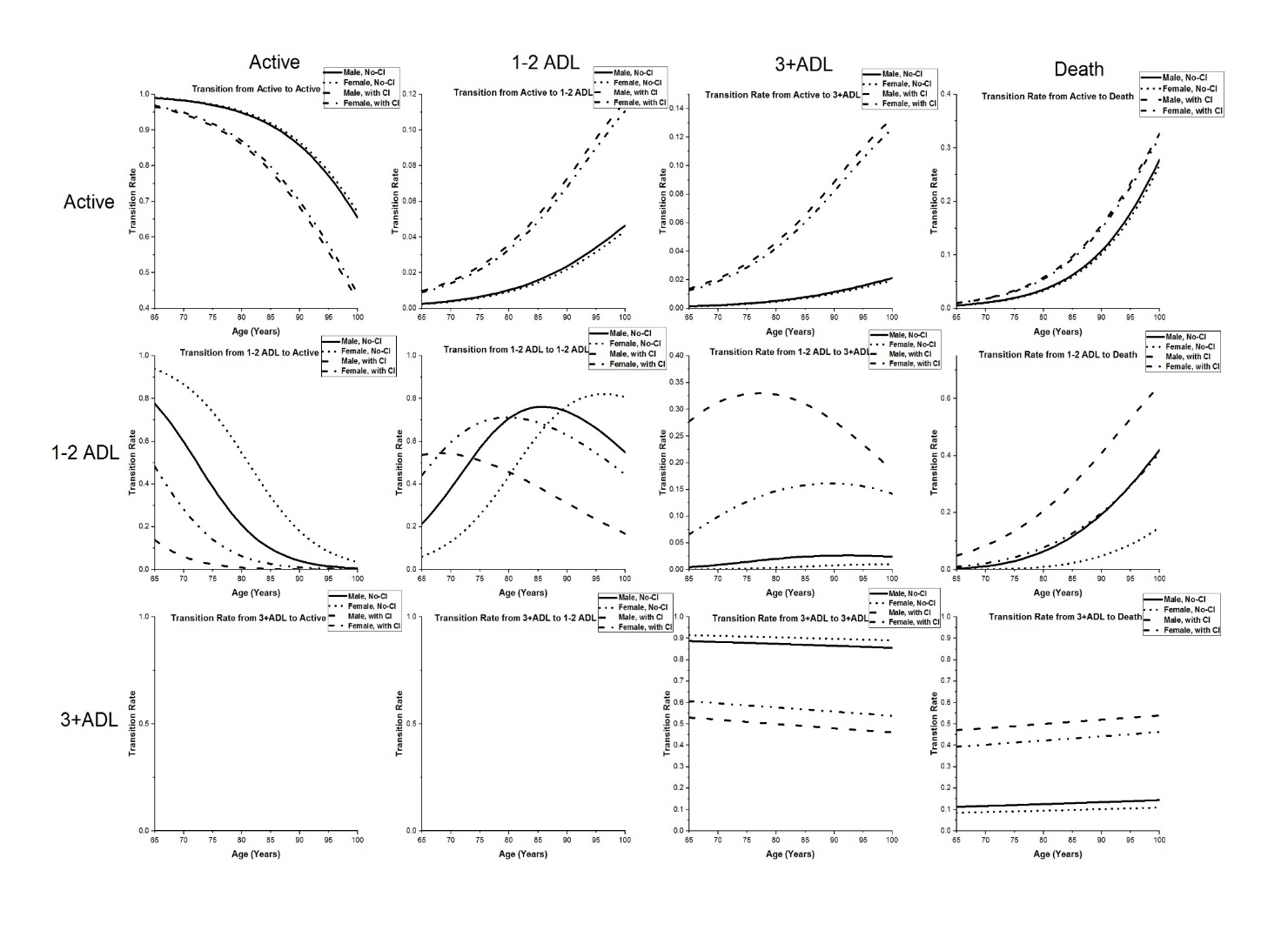


S2 Fig: Transition rates of cognitive impairment health states





**S1 Table: Projected number of older adult in China (million)**

|  | **2015** | **2030** | **2045** | **2060** |
| --- | --- | --- | --- | --- |
| **65-74 YEARS** |  |  |  |  |
| Total | 95·50 (95·46-95·53) | 156·19 (156·13-156·26) | 171·27 (171·21-171·33) | 148·18 (148·13-148·22) |
| Female | 47·44 (47·42-47·46) | 78·17 (78·13-78·20) | 85·86 (85·82-85·89) | 73·28 (73·25-73·30) |
| Male | 48·06 (48·04-48·08) | 78·03 (77·99-78·06) | 85·41 (85·38-85·45) | 74·90 (74·88-74·93) |
| **75-84 YEARS** |  |  |  |  |
| Total | 49·43 (49·39-49·48) | 100·05 (99·90-100·20) | 147·81 (147·62-148·00) | 144·83 (144·67-144·99) |
| Female | 25·50 (25·47-25·53) | 50·15 (50·05-50·25) | 74·24 (74·11-74·36) | 72·83 (72·72-72·93) |
| Male | 23·93 (23·90-23·96) | 49·90 (49·80-50·00) | 73·58 (73·45-73·70) | 72·00 (71·90-72·11) |
| **85 YEARS AND OLDER** |  |  |  |  |
| Total | 14·15 (14·211-14·19) | 41·47 (41·25-41·70) | 91·88 (91·35-92·41) | 146·34 (145·38-147·30) |
| Female | 8·23 (8·20-8·26) | 21·33 (21·18-21·48) | 47·05 (46·71-47·39) | 75·35 (74·74-75·96) |
| Male | 5·91 (5·89-5·94) | 20·14 (19·99-20·30) | 44·83 (44·47-45·19) | 70·99 (70·37-71·61) |

**S2 Table: Projected number of active older adult (older adult with no functional disability or cognitive impairment) in China (million)**

|  | **2015** | **2030** | **2045** | **2060** |
| --- | --- | --- | --- | --- |
| **65-74 YEARS** |  |  |  |  |
| Total | 92·32 (92·26-92·37) | 151·36 (151·26-151·46) | 166·10 (166·00-166·20) | 144·17 (144·00-144·24) |
| Female | 45·57 (45·54-45·60) | 75·36 (75·31-75·42) | 82·84 (82·78-82·90) | 70·93 (70·89-70·98) |
| Male | 46·74 (46·71-46·77) | 75·99 (75·94-76·05) | 83·26 (83·21-83·32) | 73·24 (73·20-73·28) |
| **75-84 YEARS** |  |  |  |  |
| Total | 40·78 (40·72-40·85) | 83·05 (82·88-83·22) | 123·87 (123·65-124·10) | 122·50 (122·30-122·70) |
| Female | 20·10 (20·06-20·14) | 40·06 (39·95-40·17) | 59·93 (59·78-60·08) | 59·40 (59·27-59·54) |
| Male | 20·68 (20·65-20·72) | 42·99 (42·87-43·11) | 63·95 (63·79-64·10) | 63·10 (62·96-63·24) |
| **85 YEARS AND OLDER** |  |  |  |  |
| Total | 8·06 (8·02-8·10) | 22·73 (22·56-22·89) | 49·74 (49·38-50·11) | 76·49 (75·90-77·08) |
| Female | 4·11 (4·08-4·13) | 10·14 (10·05-10·24) | 22·22 (22·02-22·41) | 34·18 (33·86-34·50) |
| Male | 3·95 (3·93-3·97) | 12·58 (12·46-12·70) | 27·53 (27·27-27·79) | 42·31 (41·90-42·72) |

**S3 Table: Projected number of older adult with 1-2 ADL limitations (functional disability) in China (million)**

|  | **2015** | **2030** | **2045** | **2060** |
| --- | --- | --- | --- | --- |
| **65-74 YEARS** |  |  |  |  |
| Total | 0·45 (0·43-0·47) | 0·59 (0·56-0·62) | 0·53 (0·50-0·56) | 0·36 (0·34-0·37) |
| Female | 0·20 (0·19-0·21) | 0·25 (0·24-0·26) | 0·22 (0·21-0·24) | 0·15 (0·14-0·16) |
| Male | 0·25 (0·24-0·26) | 0·34 (0·32-0·35) | 0·31 (0·29-0·32) | 0·21 (0·20-0·22) |
| **75-84 YEARS** |  |  |  |  |
| Total | 1·18 (1·15-1·20) | 2·04 (1·99-2·09) | 2·57 (2·51-2·64) | 2·16 (2·11-2·21) |
| Female | 0·61 (0·59-0·62) | 0·94 (0·91-0·97) | 1·18 (1·14-1·21) | 0·98 (0·95-1·01) |
| Male | 0·57 (0·56-0·59) | 1·10 (1·07-1·13) | 1·40 (1·35-1·44) | 1·18 (1·14-1·71) |
| **85 YEARS AND OLDER** |  |  |  |  |
| Total | 1·18 (1·16-1·20) | 3·81 (3·71-3·91) | 8·16 (7·93-8·40) | 13·21 (12·78-13·64) |
| Female | 0·79 (0·77-0·80) | 2·15 (2·08-2·21) | 4·48 (4·33-4·63) | 7·22 (6·95-7·49) |
| Male | 0·39 (0·38-0·40) | 1·66 (1·60-1·72) | 3·69 (3·54-3·84) | 6·00 (5·73-6·27) |

**S4 Table: Projected number of older adult with 3 or more ADL limitations (functional disability) in China (million)**

|  | **2015** | **2030** | **2045** | **2060** |
| --- | --- | --- | --- | --- |
| **65-74 YEARS** |  |  |  |  |
| Total | 0·65 (0·64-0·67) | 1·12 (1·09-1·15) | 1·21 (1·18-1·24) | 0·94 (0·91-0·96) |
| Female | 0·32 (0·31-0·33) | 0·56 (0·54-0·57) | 0·60 (0·58-0·61) | 0·46 (0·45-0·47) |
| Male | 0·34 (0·33-0·35) | 0·57 (0·55-0·59) | 0·61 (0·59-0·63) | 0·48 (0·46-0·49) |
| **75-84 YEARS** |  |  |  |  |
| Total | 1·47 (1·45-1·49) | 3·41 (3·34-3·47) | 4·80 (4·71-4·89) | 4·45 (4·37-4·53) |
| Female | 0·78 (0·77-0·80) | 1·79 (1·75-1·83) | 2·53 (2·47-2·58) | 2·34 (2·29-2·39) |
| Male | 0·68 (0·67-0·70) | 1·61 (1·57-1·65) | 2·27 (2·21-2·33) | 2·11 (2·05-2·16) |
| **85 YEARS AND OLDER** |  |  |  |  |
| Total | 1·54 (1·52-1·57) | 6·10 (5·96-6·24) | 14·91 (14·54-15·27) | 27·22 (26·48-27·97) |
| Female | 0·97 (0·95-0·99) | 3·52 (3·43-3·61) | 8·49 (8·27-8·72) | 15·66 (15·20-16·12) |
| Male | 0·57 (0·56-0·59) | 2·58 (2·49-2·66) | 6·41 (6·18-6·64) | 11·57 (11·12-12·01) |

**S5 Table: Projected number of older adult with mild cognitive impairment in China (million)**

|  | **2015** | **2030** | **2045** | **2060** |
| --- | --- | --- | --- | --- |
| **65-74 YEARS** |  |  |  |  |
| Total | 0·71 (0·69-0·73) | 1·19 (1·16-1·23) | 1·35 (1·31-1·39) | 1·10 (1·07-1·14) |
| Female | 0·51 (0·50-0·53) | 0·86 (0·83-0·89) | 0·98 (0·95-1·00) | 0·80 (0·77-0·82) |
| Male | 0·20 (0·19-0·21) | 0·33 (0·32-0·34) | 0·37 (0·36-0·38) | 0·31 (0·30-0·32) |
| **75-84 YEARS** |  |  |  |  |
| Total | 2·61 (2·58-2·64) | 5·14 (5·07-5·20) | 7·59 (7·50-7·68) | 7·39 (7·31-7·48) |
| Female | 1·87 (1·85-1·90) | 3·63 (3·58-3·68) | 5·38 (5·31-5·45) | 5·25 (5·18-5·32) |
| Male | 0·73 (0·72-0·75) | 1·51 (1·48-1·54) | 2·21 (2·16-2·26) | 2·14 (2·10-2·19) |
| **85 YEARS AND OLDER** |  |  |  |  |
| Total | 1·97 (1·94-1·99) | 5·98 (5·89-6·07) | 13·59 (13·36-13·81) | 22·54 (22·11-22·97) |
| Female | 1·46 (1·44-1·48) | 4·14 (4·07-4·21) | 9·36 (9·20-9·53) | 15·55 (15·25-15·86) |
| Male | 0·51 (0·50-0·52) | 1·84 (1·79-1·88) | 4·22 (4·11-4·33) | 6·99 (6·79-7·18) |

**S6 Table: Projected number of older adult with moderate cognitive impairment in China (million)**

|  | **2015** | **2030** | **2045** | **2060** |
| --- | --- | --- | --- | --- |
| **65-74 YEARS** |  |  |  |  |
| Total | 0·40 (0·39-0·40) | 0·35 (0·33-0·36) | 0·37 (0·36-0·39) | 0·29 (0·28-0·30) |
| Female | 0·25 (0·24-0·25) | 0·19 (0·18-0·19) | 0·20 (0·19-0·21) | 0·15 (0·15-0·16) |
| Male | 0·15 (0·14-0·15) | 0·16 (0·15-0·17) | 0·17 (0·16-0·18) | 0·13 (0·13-0·14) |
| **75-84 YEARS** |  |  |  |  |
| Total | 1·47 (1·45-1·48) | 1·82 (1·78-1·86) | 2·53 (2·48-2·59) | 2·36 (2·31-2·41) |
| Female | 0·96 (0·95-0·97) | 1·02 (1·00-1·05) | 1·42 (1·39-1·45) | 1·33 (1·29-1·36) |
| Male | 0·51 (0·50-0·52) | 0·80 (0·77-0·82) | 1·11 (1·07-1·15) | 1·04 (1·00-1·07) |
| **85 YEARS AND OLDER** |  |  |  |  |
| Total | 1·35 (1·34-1·37) | 2·90 (2·84-2·95) | 6·39 (6·24-6·53) | 10·91 (10·63-11·19) |
| Female | 0·97 (0·96-0·98) | 1·74 (1·70-1·79) | 3·74 (3·64-3·85) | 6·41 (6·22-6·61) |
| Male | 0·39 (0·38-0·39) | 1·15 (1·12-1·18) | 2·64 (2·56-2·72) | 4·50 (4·35-4·65) |

**S7 Table: Projected number of older adult with severe cognitive impairment in China (million)**

|  | **2015** | **2030** | **2045** | **2060** |
| --- | --- | --- | --- | --- |
| **65-74 YEARS** |  |  |  |  |
| Total | 1·14 (1·11-1·16) | 1·81 (1·76-1·85) | 1·94 (1·89-1·99) | 1·49 (1·45-1·53) |
| Female | 0·70 (0·68-0·71) | 1·08 (1·05-1·11) | 1·16 (1·13-1·19) | 0·89 (0·86-0·91) |
| Male | 0·44 (0·43-0·45) | 0·72 (0·70-0·75) | 0·78 (0·75-0·80) | 0·60 (0·58-0·62) |
| **75-84 YEARS** |  |  |  |  |
| Total | 2·98 (2·95-3·01) | 6·55 (6·47-6·64) | 9·13 (9·01-9·25) | 8·41 (8·31-8·52) |
| Female | 1·90 (1·87-1·92) | 3·95 (3·89-4·02) | 5·50 (5·42-5·59) | 5·07 (4·98-5·15) |
| Male | 1·08 (1·06-1·10) | 2·60 (2·55-2·65) | 3·63 (3·56-3·69) | 3·35 (3·29-3·41) |
| **85 YEARS AND OLDER** |  |  |  |  |
| Total | 1·62 (1·61-1·64) | 5·10 (5·02-5·18) | 11·19 (11·00-11·38) | 17·70 (17·35-18·05) |
| Female | 1·13 (1·12-1·14) | 3·13 (3·08-3·19) | 6·82 (6·70-6·94) | 10·80 (10·59-11·02) |
| Male | 0·49 (0·48-0·50) | 1·96 (1·92-2·01) | 4·37 (4·26-4·48) | 6·90 (6·70-7·10) |

**RESEARCH IN CONTEXT**

From October 24^th^, 2019 to November 1^st^, 2019, we searched PubMed and Web of Science for studies projecting future trends of functional and cognitive disability among the elderly in China, with the search terms ("projecting*", OR "trends*" OR "forecast*") AND ("functional disability*" OR "cognitive impairment*" OR "disability*") AND ("elderly*" OR "older adult*") AND ("china"). We identified 160 papers from PubMed and 342 papers from Web of Science. A review of abstracts identified relevant articles which were then extracted. We identified only eight papers forecasting trends in functional disability or cognitive impairment or dementia in China. None of these studies projects future trends in disability—functional and cognitive—using empirically estimated transition rates in a dynamic model; as well as, accounting for the impact of cognitive impairment on the prevalence of functional disability. The list of the eight papers found are provided in the appendix.

**S8 Table: Results of literature review on projection of functional and cognitive disability in China**

| **Author** | **Title** | **Population** | **Age** | **Time Horizon** | **Outcomes** | **Factors Included** |
| --- | --- | --- | --- | --- | --- | --- |
| Ansah et. Al^4^ | Projecting the Number of Elderly with Cognitive  Impairment in China Using a Multi-State  Dynamic Population Model | China | 65+ | Up to 2060 | Prevalence of Cognitive Impairment (Mild, Moderate, Severe) | Age, Sex |
| Hanewald et. al.^5^ | Modelling multi-state health transitions in China:  a generalised linear model with time trends | China | 65-105 | 1998 – 2012 | Mortality rates / Disability rates; Life expectancy / Healthy life expectancy | Age, time, sex, urban/rural residence |
| Hu et. al.^6^ | Projecting future demand for informal care  among older people in China: the road  towards a sustainable long-term  care system | China |  | 2015 - 2035 | Number of informal care recipients required (based on source, type, and intensity of care) | Age, gender, severity of disability, rural-urban residence |
| Liu et. al.^7^ | Are China’s oldest-old living longer with  less disability? A longitudinal modeling  analysis of birth cohorts born 10 years  apart | China | 80+ | N.A. | Disability, disability free life expectancy | Age, sex, education, rural-urban residence |
| Woo et. al.^8^ | An estimation of the functional disability  burden in elderly Chinese age 70 years and over | Hong Kong | 70+ | N.A. | Disability years, disability ADL | Age, sex |
| Yu et. al.^9^ | Multi-state Markov model in outcome of mild cognitive  impairments among community elderly resident’s in  Mainland China | China | 65+ | 2006-2011 | Alzheimer’s disease | Age, sex, education, hypertension, diabetes, reading, APOEε4 allele |
| Yu et. al.^10^ | A comparison of health expectancies over 10 years: implications  for elderly service needs in Hong Kong | Hong Kong | 65+ | 2021-2041 | Physical, cognitive impairment; number of carers needed | Age, period, marital status, educational attainment, hearing and eyesight problems |
| Zheng et. al.^11^ | Implications of Changes  in Households and Living  Arrangements for Future  Home-Based Care Needs  and Costs for Disabled  Elders in China | China | 65+ | 2010-2050 | Disability, Disability ADL | Age, sex, rural-urban residence, marital status, co-residence with children |

**REFERENCES:**

1. Kryscio RJ, Schmitt FA, Salazar JC, Mendiondo MS, Markesbery WR. Risk factors for transitions from normal to mild cognitive impairment and dementia. *Neurology*. 2006;**66**(6):828–32.
2. Tyas SL, Salazar JC, Snowdon DA, Desrosiers MF, Riley KP, Mendiondo MS, et al. Transitions to mild cognitive impairments, dementia, and death: findings from the Nun Study. *Am J Epidemiol*. 2007;**165**(11):1231–8.
3. Cai L, Hayward M, Saito Y, Lubitz J, Hagedorn A, Crimmins E. Estimation of multi-state life table functions and their variability from complex survey data using the SPACE Program. Demographic Research. 2010;**22**(6):129–58.
4. Ansah, J. P., Koh, V., Chiu, C. , Chei, C. , Zeng, Y. , Yin, Z. , Shi, X. and Matchar, D. B. Projecting the Number of Elderly with Cognitive Impairment in China Using a Multi‐State Dynamic Population Model. *Syst. Dyn. Rev.* 2017;33: 89-111. doi:10.1002/sdr.1581
5. Hanewald K, Li H, Shao AW. Modelling multi-state health transitions in China: a generalised linear model with time trends. *Annals of Actuarial Science*. 2019;13(1):145-165. doi:10.1017/S1748499518000167
6. Hu B. Projecting future demand for informal care among older people in China: the road towards a sustainable long-term care system. *Health Economics, Policy and Law*. 2019;14(1):61-81. doi:10.1017/S1744133118000221
7. Liu, Z., Han, L., Feng, Q. et al. Are China’s oldest-old living longer with less disability? A longitudinal modeling analysis of birth cohorts born 10 years apart. *BMC Med.* 2019; 17(23). doi:10.1186/s12916-019-1259-z
8. J. Woo, S. C. Ho, Y. K. Yuen, L. M. Yu & J. Lau. An estimation of the functional disability burden in elderly Chinese age 70 years and over, *Disability and Rehabilitation*. 1996;18(12) 609-612. DOI: 10.3109/09638289609166322
9. Yu H-Mei, Yang S-Shan, Gao J-Wei, Zhou L-ye, Liang R-Feng, Qu C-Yi. Multi-state Markov model in outcome of mild cognitive impairments among community elderly residents in Mainland China. International Psychogeriatrics. 2013;25(5):797-804. doi:10.1017/S1041610212002220
10. Yu, R., Leung, J., Lum, C.M. et al. A comparison of health expectancies over 10 years: implications for elderly service needs in Hong Kong. *Int J Public Health*. 2019;64: 731. https://doi.org/10.1007/s00038-019-01240-1
11. Zeng, Y., Chen, H., Wang, Z., & Land, K.C. Implications of changes in households and living arrangements for future home-based care needs and costs for disabled elders in China. Journal of aging and health, 2015; 27(3), 519-50. doi: 10.1177/0898264314552690
